# Safety and Prophylactic Efficacy of Liposome-Based Vaccine against the Drug-Resistant *Acinetobacter baumannii* in Mice

**DOI:** 10.3390/pharmaceutics14071357

**Published:** 2022-06-27

**Authors:** Masood Alam Khan, Khaled S. Allemailem, Hamzah Maswadeh, Hina Younus

**Affiliations:** 1 Department of Basic Health Sciences, College of Applied Medical Sciences, Qassim University, Buraydah 51452, Saudi Arabia; 2 Department of Medical Laboratories, College of Applied Medical Sciences, Qassim University, Buraydah 51452, Saudi Arabia; k.allemailem@qu.edu.sa; 3 Department of Pharmaceutics, College of Pharmacy, Qassim University, Buraydah 51452, Saudi Arabia; msodh@qu.edu.sa; 4 Interdisciplinary Biotechnology Unit, Aligarh Muslim University, Aligarh 202002, India; hinayounus@rediffmail.com

**Keywords:** *Acinetobacter baumannii*, liposomes, immunization, immune response

## Abstract

In recent years, the emergence of multidrug-resistant *Acientobacter baumannii* has greatly threatened public health and depleted our currently available antibacterial armory. Due to limited therapeutic options, the development of an effective vaccine formulation becomes critical in order to fight this drug-resistant pathogen. The objective of the present study was to develop a safe vaccine formulation that can be effective against *A. baumannii* infection and its associated complications. Here, we prepared liposomes-encapsulated whole cell antigens (Lip-WCAgs) as a vaccine formulation and investigated its prophylactic efficacy against the systemic infection of *A. baumannii*. The immunization with Lip-WCAgs induced the higher production of antigen-specific antibody titers, greater lymphocyte proliferation, and increased secretion of Th1 cytokines, particularly IFN-γ and IL-12. Antisera from Lip-WCAgs-immunized mice showed the utmost bactericidal activity and potently inhibited the biofilm formation by *A. baumannii*. Interestingly, Lip-WCAgs-induced immune response was translated in in vivo protection studies as the immunized mice exhibited the highest resistance to *A. baumannii* infection. Mice in the group immunized with Lip-WCAgs had an 80% survival rate and a bacterial burden of 5464 ± 1193 CFUs per gram of the lung tissue, whereas the mice immunized with IFA-WCAgs had a 50% survival rate and 51,521 ± 8066 CFUs. In addition, Lip-WCAgs vaccinated mice had lower levels of the inflammatory markers, including CRP, IL-6, IL-1β, and TNF-α. The findings of this study suggest that Lip-WCAgs may be considered a potential vaccine formulation to protect individuals against *A. baumannii* infection.

## 1. Introduction

*Acinetobacter baumannii* is a gram-negative coccobacillus that mostly occurs as a commensal on the skin, soil, and health care facilities [1,2]. In the last few decades, *A. baumannii* has emerged as a life-threatening opportunistic pathogen in health care settings. The hospitals in the Kingdom of Saudi Arabia are showing a continuous upsurge in the emergence of antibiotic-resistant *A. baumannii*, which is becoming a major health care burden [3,4]. *A. baumannii* is a multifaceted pathogen that causes infections of the respiratory tract, urinary tract, bloodstream, and skin [5]. *A. baumannii*-associated pneumonia accounts for about a 35–70% mortality rate [3]. Immunosuppression, respiratory failure, and prior antimicrobial therapy constitute common risk factors for *A. baumannii* infection [3]. The frequency of multidrug-resistant *A. baumannii* has been increased to 83.6% for ceftriaxone, 82% for piperacillin-tazobactam, and 80.3% for ceftazidime in Saudi Arabia [6,7]. The formation of the biofilm is an important tactic adopted by *A. baumannii* in order to counter the effects of antibiotics, the host’s immune response, and adverse environmental conditions [8]. The biofilm formation renders antibiotics ineffective, which results in the emergence of antibiotic-resistant *A. baumannii*. Moreover, multidrug-resistant (MDR) *A. baumannii* has been often isolated from immunocompromised persons who received broad-spectrum antibiotics [9,10]. How to combat the MDR isolates of *A. baumannii* is a major challenge to clinicians because of the chronic nature of these infections.

Currently, there is no vaccine available that can be used to control *A. baumannii* and its infection-associated pneumonia. Hence, it is momentous to develop a safe and effective vaccine that can prevent *A. baumannii* infection in humans. Earlier, many researchers have tried to develop a reliable vaccine against *A. baumannii*. Various forms of *A. baumannii* antigens have been exploited to formulate the best possible vaccine, including DNA vaccines and inactivated whole organism and outer membrane protein A (OmpA)-based vaccines [11]. The subunit and recombinant vaccine using Omp A as an antigen can induce the protective immune responses against the drug-resistant *A. baumannii* [12,13]. Du et al., demonstrated that the immunization with synthesized multi-epitope polypeptide rOmp22 encapsulated in chitosan-PLGA nanoparticles induced higher levels of antigen-specific immune response and protected mice against *A. baumannii* infection [14].

Liposomes are vesicles that are composed of amphiphilic phospholipids, such as phosphatidylcholine, phosphatidylserine, and sphingomyelin. We have earlier reviewed that the surface modification of liposomes can increase their prophylactic and therapeutic effectiveness [15,16]. Recently, a study by Alameh et al., reported that lipid nanoparticles increased the efficacy of protein and mRNA vaccines by inducing the strong humoral and T follicular helper cell responses [17]. A liposomal vaccine formulation of receptor-binding domain-encoding mRNA (RBD-mRNA) induced superior immunogenicity in mice and protected the host cells against SARS-CoV-2 infection [18]. Liposomal vaccines have also been reported to be effective against bacterial infections as well. A liposomal formulation of adjuvant DMT (consisting of dimethyldioctadecylammonium, monophosphoryl lipid A, and trehalose 6,6′-dibehenate) enhanced the prophylactic efficacy of the DNA vaccine against *Mycobacterium tuberculosis* infection in mice [19]. Bhalla et al., demonstrated that a liposomal formulation of polysaccharides successfully protected aged mice against pulmonary pneumococcal infection [20]. Liposomes composed of *E. coli* lipids (Escheriosomes) and loaded with *Salmonella typhimurium* antigens induced the generation of the cytotoxic T lymphocyte (CTL) responses that effectively protected mice against bacterial infection [21]. The superior immunoadjuvant potential of liposomes can be inferred from their unique property of they can deliver the encapsulated antigens to the intracellular compartments of antigen-presenting cells (APCs) that can induce both CD4+ and CD8+ T cells [22]. None of the earlier studies showed the prophylactic efficacy of the liposome-based vaccine against *A. baumannii* infection in model animals. The present work aimed to develop an effective and safe vaccine that can successfully protect individuals against *A. baumannii* without inducing any significant toxic manifestations. Here, we prepared liposome-encapsulated whole cell antigens (Lip-WCAgs) of *A. baumannii* and investigated their prophylactic efficacy against *A. baumannii* infection in a murine model.

## 2. Materials and Methods

### 2.1. Materials

Liposome-grade 1,2-dipalmitoyl-Sn-glycerol-3-phosphocholine (DPPC) and cholesterol (Chol) were obtained from the Avanti Polar Lipids (Alabaster, AL, USA). Serum alanine transaminase (ALT), aspartate transaminase (AST), blood urea nitrogen (BUN), and lactate dehydrogenase (LDH) kits were purchased from Quimica Clinica Aplicada, Amposta, Tarragona, Spain. The ELISA kits for cytokines and inflammatory markers were purchased from Abcam (Cambridge, UK). The cell proliferation assay kit was bought from Abcam, Cambridge, UK. The isotypes analysis kits were purchased from Sino Biologicals Inc. (Beijing, China).

### 2.2. Mice

Female BALB/C mice (10–12 weeks) of an average weight of 25 ± 5 g were used in the study and were given a standard pellet diet (Hindustan Lever Ltd., Mumbai, India) and water ad libitum. The techniques used for bleeding, injection, as well as the sacrifice of mice, were performed after approval from the Animal Ethics Committee of the College of Applied Medical Sciences, Qassim University, Buraydah, Saudi Arabia.

### 2.3. Preparation of Whole Cell Antigens (WCAgs) from A. baumannii

A multidrug-resistant strain of *A. baumannii* (ATCC 19606) was cultured in nutrient broth and the bacterial cells were collected by centrifugation. The cell pellet was washed and lysed in lysis buffer containing 0.75 M sucrose, 10 mM Tris-HCl, 10 mg/mL of lysozyme, and 1.5 mM EDTA, containing a protease inhibitor cocktail. The sample was sonicated followed by centrifugation at 5000 rpm in order to collect the supernatant. The concentration of the protein was determined by the bicinchoninic acid (BCA) method as described earlier [23].

### 2.4. Preparation and Characterization of Liposomes

Liposomes were prepared from dipalmitoylphosphatidylcholine (DPPC) and cholesterol [23]. Briefly, the mixture of DPPC and cholesterol was dissolved in chloroform/methanol (1:1) that was evaporated to form a thin lipid film. The dried lipid film was hydrated with WCAgs solution and was flash-frozen. The frozen mixture was lyophilized and was later rehydrated. The dried, reconstituted vesicles were centrifuged at 10,000 rpm for 15 min at 4 °C. The pellet was separated and the amount of the encapsulated antigen was measured by the BCA method. The percentage of encapsulated antigen was found to be 54%.

The size, polydispersity index (PDI), and zeta potential of the liposomes were determined by the Malvern Nano Zeta Sizer (Malvern Instruments, Southborough, MA, USA) by using the dynamic light scattering (DLS) technique [24].

### 2.5. Mice

Female BALB/C mice of 12 weeks were taken from the animal house facility of the College of Applied Medical Sciences, Qassim University, Saudi Arabia. The experiments were approved by the committee of research ethics, Deanship of Scientific Research, Qassim University, Buraydah, Saudi Arabia.

### 2.6. Immunization of Mice with Liposome-Encapsulated Acinetobacter baumannii Whole Cell Antigens (WCAgs)

Each mouse was subcutaneously immunized with a dose of 10 μg WCAgs in various immunoadjuvant formulations, including incomplete Freund’s adjuvant (IFA) and liposomes. The same amount of free antigen was injected into the mice of the control group. Further, each group of mice received two booster doses on days 14 and 21. Mice were divided into five following groups and each group contained 10 mice.

1. PBS
2. Sham Liposomes
3. Free WCAgs
4. IFA-WCAgs
5. Lip-WCAgs

### 2.7. Evaluation of the Safety of Vaccine Formulations

The blood was collected from the mice through retro-orbital puncture on day 5 after the final booster dose. In order to determine the safety of the vaccine, the levels of ALT, AST, BUN, and LDH were analyzed in the blood of control and immunized mice [24].

### 2.8. Determination of the IgG Titer and IgG Isotyping

The titers of antigen-specific IgG, IgG1, and IgG2a were measured by ELISA as described earlier [23]. Briefly, WCAgs (5 µg/mL) was coated into the wells of Maxisorp ELISA plates (NUNC, Roskilde, Denmark) overnight at 4 °C. The plates were washed and the wells were blocked with 5% skimmed milk. Following the extensive washing, the plates were incubated with serial dilutions of serum for 2 h. The plates were washed and rabbit anti-mouse IgG-HRP was added for 1 h. Finally, the reaction was developed by adding 100 µL of 3,3′,5,5′-tetramethylbenzidine (TMB), and the absorbance was recorded at 450 nm.

### 2.9. Determination of the Proliferation of Lymphocytes

The proliferation assay was performed in the splenocytes using the cell titer 96 nonradioactive BrdU calorimetric ELISA kit from Abcam, Cambridge, UK. The spleen was excised from the immunized mice and the splenocytes (1 × 10^5^) were stimulated with WCAgs (0.01 to 10 µg/mL) for 48 h. The cells stimulated with Con A acted as a positive control, whereas the splenocytes stimulated with ovalbumin were used as a negative control. After 48 h, the cells were incubated with the diluted BrdU (20 µL), centrifuged and the fixing solution was added to them for 30 min, and after washing, anti-BrdU was added for 1 h. After washing, peroxidase-conjugated goat anti-mouse IgG was added, followed by the addition of 100 µL of TMB substrate. Finally, the reaction was stopped by adding 100 µL of the stop solution. The absorbance was taken at a dual wavelength of 450/550 nm. The simulation index was calculated as follows: mean of the absorbance of the test/mean of the absorbance of the negative control.

### 2.10. Analysis of Cytokines

The splenocytes (1 × 10^6^) were stimulated with 10 µg/mL of WCAgs and incubated for 48 h at 37 °C [23]. As a control, the splenocytes were treated with 10 µg/mL of ovalbumin. The supernatant was collected to determine the levels of IFN-γ, IL-4, and IL-12.

### 2.11. Determination of the Opsonophagocytic Killing Activity of Serum

*A. baumannii* (ATCC 19606) was grown in tryptic soy broth (TSB) at 37 °C for 24 h. Murine RAW 264.7 macrophage cells were cultured in RPMI 1640 supplemented with 10% fetal bovine serum (FBS), 1% penicillin, streptomycin, glutamine, and 50 µM β-mercaptoethanol as described earlier [25]. The cells were activated with PMA for 3 days. Macrophages (2 × 10^6^ cells) and *A. baumannii* (1 × 10^5^ cells) in a 20:1 ratio were taken into each well. The antisera from unimmunized and immunized mice were heated at 56 °C for 30 min for complement inactivation. The antiserum was diluted in 1:1, 1:10, 1:100, and 1:1000 ratios with a culture medium and mixed with macrophages and bacteria for an hour. Aliquots of the suspension (10 µL) were taken from each well and were plated on TSB plates in order to determine the number of colony-forming units (CFUs). The percentage of killing was determined by using the following formula: % bacteria killed = (1 − [bacteria survived in the presence of antisera/bacteria survived in negative control sera]) × 100.

### 2.12. Determination of the Effect of Antisera on the Biofilm Formation by A. baumannii

The efficacy of the antisera was evaluated against the biofilm formation by *A. baumannii* [26]. *A. baumannii* (1 × 10^6^ CFUs/mL) was taken in the TSB and was incubated with 10-fold diluted antisera from the immunized mice. After incubation for 24 h, the plate was washed with sterile PBS. The wells were dried and 0.1% crystal violet solution was added to each well. After washing, the plate was dried and the remaining stain was extracted by adding 150 µL of ethanol. Finally, the absorbance of the content was taken at 595 nm by using the microplate spectrophotometer.

### 2.13. Mouse Model of A. baumannii Infection

*A. baumannii* cells were grown in TSB at 37 °C and were centrifuged at 5000 rpm for 15 min at 4 °C. The cell pellet was washed twice with PBS. Each mouse was intravenously infected with 5 × 10^6^ CFUs of *A. baumannii* two weeks after the final booster dose.

### 2.14. Assessment of the Prophylactic Potential of Vaccine Formulations against A. baumannii

The effectiveness of the immunization against *A. baumannii* infection was analyzed by observing the survival and determining the bacterial load in the lung tissues [27]. The mice were checked daily for 30 days post-infection in order to analyze their survival. For the assessment of the bacterial load, three mice from each group were sacrificed on day 5 post-*A. baumannii* infection. The lungs were removed and homogenized in cold-PBS and various dilutions of tissue homogenate were spread on nutrient agar plates. After 24 h, the CFUs were counted and multiplied by the dilution factor.

### 2.15. Determination of Inflammation Markers in the Systemic Circulation

In order to evaluate the efficacy of vaccine formulations against infection-induced inflammation, the levels of the C-reactive protein (CRP), IL-6, IL-1β, and TNF-α were measured in the blood of mice [27].

### 2.16. Statistical Analyses

The survival rate of the mice was analyzed by the Kaplan–Meier curve by using the Log-rank chi square test. The data of the bacterial load were analyzed by one-way ANOVA and Turkey post-test by using the GraphPad Prism software, version 5.0 (La Jolla, CA, USA). The data are represented as the mean ± S.D. of all experimental values.

## 3. Results

### 3.1. PDI, Size, and Zeta Potential of Liposomes

The PDI value of the liposomes was found to be 0.328, whereas the mean size was found to be 176 nm as measured by DLS. The zeta potential of liposomes was −12.4 mV.

### 3.2. Immunization with Lip-WCAgs Did Not Induce Any Remarkable Toxicity in Mice

**Figure 1:**
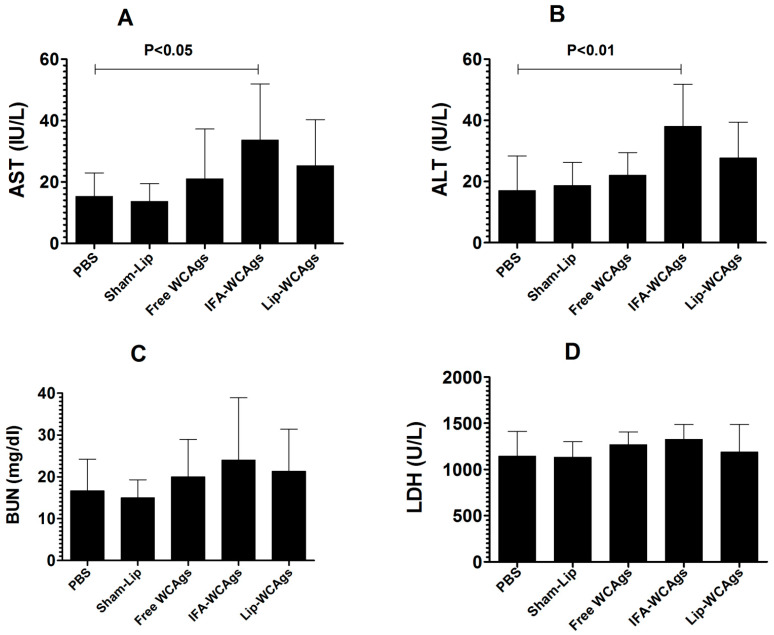
Lip-WCAgs vaccine formulation was safe and did not induce any toxicity in mice. The (**A**) AST level, (*p* < 0.05) PBS vs. IFA-WCAgs, (**B**) ALT level, *p* < 0.01, PBS vs. IFA-WCAgs, (**C**) BUN level, and (**D**) LDH in immunized mice. The data are mean ± SD of three experiments.

The toxicity of vaccine formulation in the mice was assessed by analyzing the levels of AST, ALT, BUN, and LDH in the blood of immunized mice. Mice immunized with Lip-WCAgs did not show any considerable elevation in the levels of AST, ALT, BUN, and LDH in the blood (Figure 1). The level of AST was found to be 15.3 ± 3.1 IU/L in the PBS-injected mice, whereas the AST level was increased to 33.67 ± 7.3 IU/L in the mice immunized with IFA-WCAgs (*p* < 0.05) (Figure 1A). However, the mice immunized with Lip-WCAgs had a 20.33 ± 6 IU/L AST level. The ALT level was found to be 17 ± 4.5 IU/L in PBS-injected mice. The mice immunized with IFA-WCAgs showed an ALT level of 38 ± 5.5 IU/L, whereas those immunized with Lip-WCAgs had an ALT level of 23 ± 3.6 IU/L (Figure 1B). The effect of immunization on renal toxicity was assessed by determining the level of BUN. The BUN levels were found to be 16.67 ± 3 in the blood of mice injected with PBS (Figure 1C). Nevertheless, the BUN level was significantly increased to 24 ± 6 mg/dL in the mice immunized with IFA-WCAgs. Importantly, the mice immunized with Lip-WCAgs had a BUN level of 17.7 ± 4 mg/dL. Similarly, the LDH level in the mice injected with PBS was 1143 ± 107 U/L, which was increased to 1328 ± 64 U/L in the mice immunized with IFA-WCAgs (Figure 1D). The mice immunized with Lip-WCAgs had an LDH level of 1190 ± 120 U/L (Figure 1D).

### 3.3. Immunization with Lip-WCAgs Induced the Greater the Secretion of Total IgG and IgG2a

**Figure 2:**
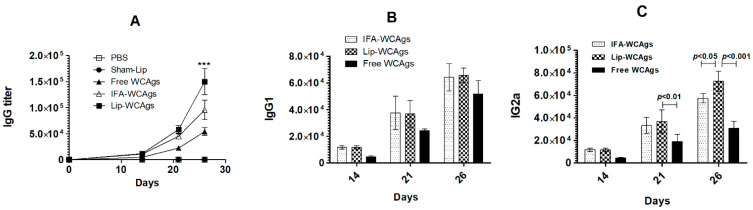
Mice immunized with Lip-WCAgs had higher levels of serum IgG and IgG2a. The levels of (**A**) IgG, *** (*p* < 0.001) Lip-WCAgs vs. IFA-WCAgs (**B**) IgG1, and (**C**) IgG2a on days 14, 21, and 28 post-immunization. The data are expressed as mean ± SD of three values.

The sera of the mice immunized with Lip-WCAgs had the highest level of antigen-specific IgG titer as compared to that in mice immunized with IFA-WCAgs or Free WCAgs, particularly on day 26 (Figure 2A) (*p* < 0.001). The mice in the group immunized with Free WCAgs (without any adjuvant) showed the lowest level of IgG titer. The outcome of the immunization with Free WCAgs or IFA-WCAgs or Lip-WCAgs was also assessed on the generation of IgG isotypes, including IgG1 and IgG2a. There was no significant difference in the levels of serum IgG1 between the mice immunized with IFA-WCAgs and Lip-WCAgs (Figure 2B). Interestingly, Lip-WCAgs-immunized mice had significantly augmented levels of IgG2a as compared to those immunized with Free WCAgs or IFA-WCAgs (*p* < 0.001 and *p* < 0.05, respectively) (Figure 2C). On day 26 of immunization, the level of IgG2a was found to be 72,500 ± 5204 as compared to 57,500 ± 2500 and 30,833 ± 3632 in the mice from the groups immunized with IFA-WCAgs and Free WCAgs, respectively.

### 3.4. Splenocytes from Mice Immunized with Lip-WCAgs Showed Greater Proliferation

**Figure 3:**
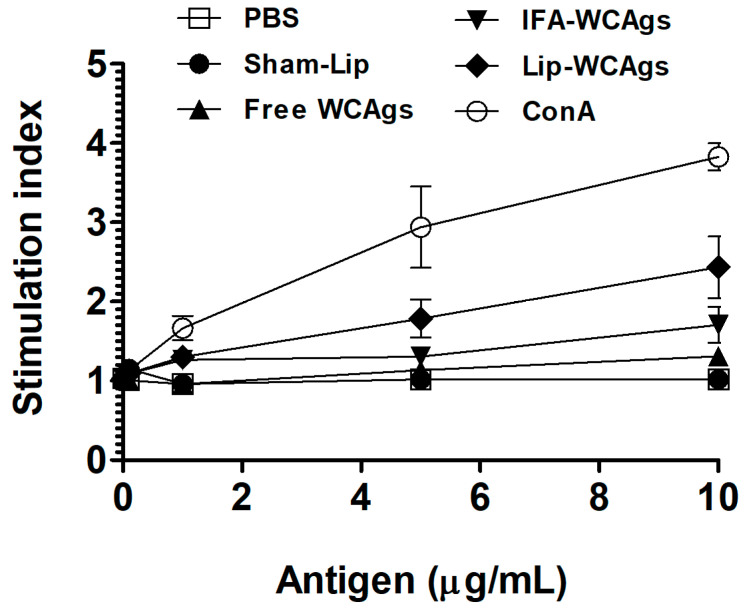
The splenocytes from Lip-WCAgs-immunized mice demonstrated the greatest proliferation in response to WCAgs. The values are expressed as the mean ± SD of three experiments.

The splenocytes from mice immunized with Lip-WCAgs showed remarkably greater proliferation with a stimulation index of 2.43 ± 0.226 as compared to the stimulation indices of 1.3 ± 0.5 and 1.7 ± 0.13 of the splenocytes from Free WCAgs and IFA-WCAgs-immunized mice, respectively (Figure 3). The mice injected with Sham-lip or PBS did not show any increase in lymphocyte proliferation.

### 3.5. The Splenocytes from the Mice Immunized with Lip-WCAgs Secreted Higher Levels of Cytokines

**Figure 4:**
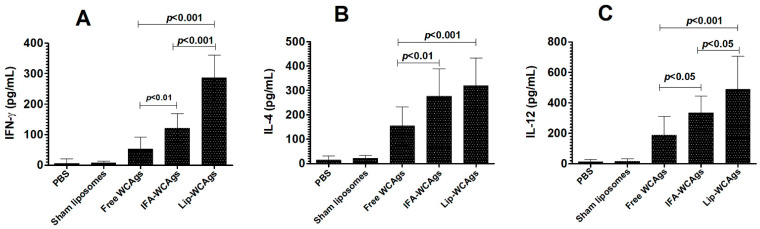
The splenocytes from Lip-WCAgs-immunized mice secreted larger IFN-γ and IL-12, but not IL-4. (**A**) IFN-γ, (**B**) IL-4, and (**C**) IL-12. The spleen cells were stimulated with WCAgs (10 µg/mL) for 48 h. The supernatant was collected and the levels above-mentioned cytokines were analyzed. The data are expressed as mean ± SD.

The secretion of IFN-γ, IL-4, and IL-12 was analyzed in the culture supernatant of splenocytes from mice immunized with Lip-WCAgs or Free WCAgs (Figure 4). The splenocytes from the mice immunized with Lip-WCAgs produced 286 ± 30 pg/mL of IFN-γ, whereas the mice immunized with IFA-WCAgs secreted 121 ± 19 pg/mL of IFN-γ (Figure 4A) (*p* < 0.001). Immunization with Free WCAgs was the least effective in inducing the secretion of IFN-γ as the splenocytes from the mice immunized with Free WCAgs produced 53 ± 16 pg/mL of IFN-γ. In addition, the production of IL-4 was analyzed in the culture supernatant of the splenocytes. The splenocytes from the mice immunized with Lip-WCAgs produced 319 ± 45 pg/mL of IL-4 (Figure 4B). The splenocytes from the mice immunized with IFA-WCAgs secreted IL-4 levels of 276 ±45 pg/mL, whereas Free WCAgs produced 154 ± 31 pg/mL of IL-4 (Figure 4B). The level of IL-12 was also quantified in the culture supernatants of the splenocytes from Free WCAgs or IFA-WCAgs or Lip-WCAgs-immunized mice (Figure 4C). The culture supernatant from the splenocytes from Lip-WCAgs-immunized mice had an IL-12 level of 488 ± 88 pg/mL, which was significantly higher than the 334 ± 44 pg/mL of IL-12 secreted by the splenocytes from IFA-WCAgs-immunized mice (*p* < 0.05). However, the secretion of IL-12 was found to be 187 ± 51 pg/mL by the splenocytes from the mice immunized with Free WCAgs (Figure 4C). The splenocytes from the mice injected with PBS or Sham-Lip produced insignificant amounts of IL-12.

### 3.6. Antisera from Lip-WCAgs-Immunized Mice Showed Greater Opsonophagocytic Killing of A. baumannii

**Figure 5:**
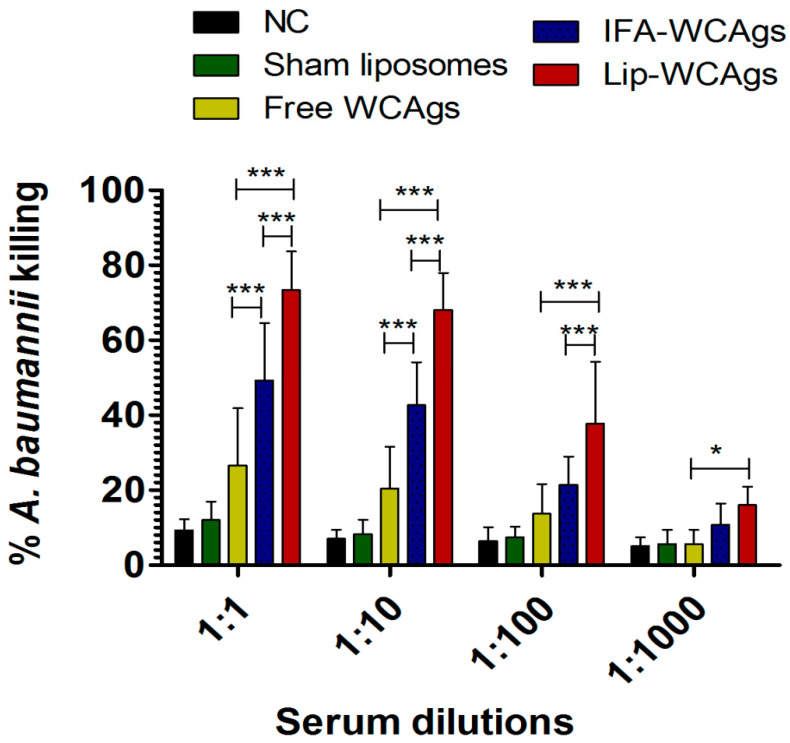
Antiserum from Lip-WCAgs-immunized mice showed the highest bactericidal activity against *A. baumannii*. The antiserum was diluted with a culture medium and mixed with macrophages and bacteria for one hour. The aliquots (10 µL) were taken and plated on TSB plates to determine the numbers of CFUs. The data are mean ± SD of three different experiments. *** *p* < 0.001, * *p* < 0.05.

The opsonophagocytic activity of antisera from the immunized mice was analyzed against *A. baumannii*. The findings showed that antiserum from Lip-WCAgs-immunized mice showed the highest bactericidal activity in the presence of macrophages. At a dilution of 1:1, antiserum from Lip-WCAgs-immunized mice demonstrated 73.3 ± 5.7% killing which was significantly greater than the 49 ± 12.3% killing in the presence of antiserum from IFA-WCAgs-immunized mice (Figure 5) (*p* < 0.001). Antiserum from the mice immunized with Free WCAgs showed only 26.7 ± 6.1% killing of *A. baumannii* at the same dilution (Figure 5). Interestingly, the antiserum from Lip-WCAgs-immunized mice showed greater opsonophagocytic killing at the dilutions of 1:10 and 1:100 (Figure 5).

### 3.7. A. baumannii-Specific Antisera Inhibited the Biofilm Formation

**Figure 6:**
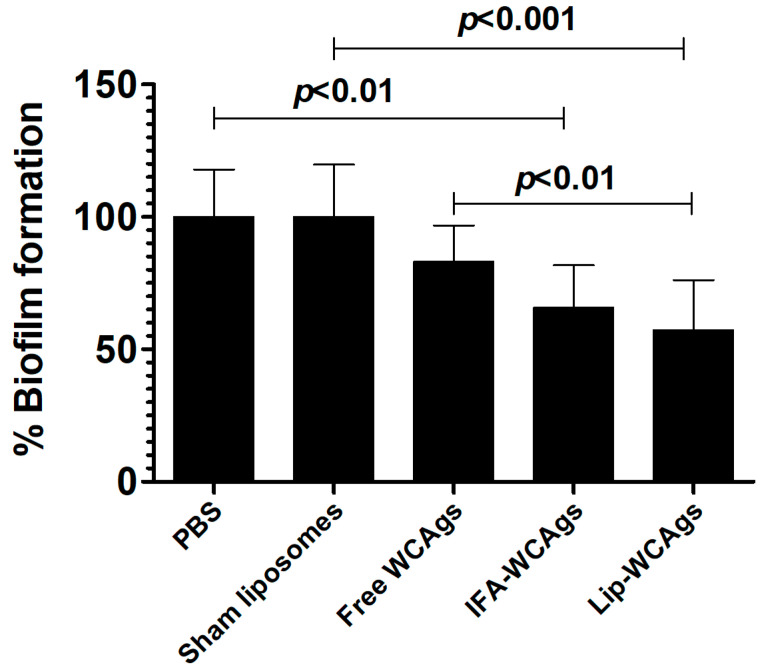
Antiserum from Lip-WCAgs-immunized mice showed the largest inhibition in biofilm formation. *A. baumannii* (1 × 10^6^ CFUs/mL) was incubated with 10-fold diluted antisera for 24 h. After washing, the wells were dried and 0.1% crystal violet solution was added for 15 min. After washing, 50 µL of ethanol was added to extract the stain and the absorbance of the content was taken at 595 nm. The data are expressed mean ± SD of three values.

We tested the efficacy of the antisera against the formation of biofilm by *A. baumannii* (Figure 6). The treatment with antisera from the mice immunized with Lip-WCAgs reduced the biofilm formation to 57% as compared to that in the presence of antisera from Sham-Lip injected mice (Figure 6) (*p* < 0.001). Whereas, the biofilm formation in the presence of the antisera from the mice immunized with IFA-WCAgs was reduced to 66% (Figure 6) (*p* < 0.01). However, the biofilm formation in the presence of antisera from the mice immunized with Free WCAgs was marginally reduced to 83%.

### 3.8. Immunization with Lip-WCAgs Effectively Protected the Mice against the Systemic Infection of A. baumannii

**Figure 7:**
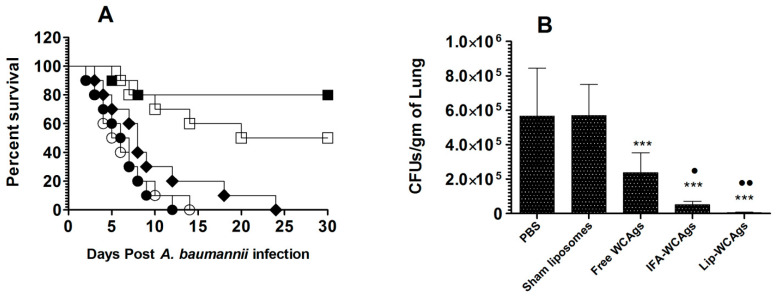
Mice immunized with Lip-WCAgs showed the highest resistance against the systemic infection of *A. baumannii*. (**A**) Each mouse from the immunized groups was intravenously infected with 5 × 10^6^ cells with *A. baumannii* and was observed for 30 days. PBS (●), Sham liposomes (○), Free WCAgs (♦), IFA-WCAgs (☐), Lip-WCAgs (■). (**B**) The bacterial burden was assessed as mentioned in the methods. *** *p* < 0.001, PBS vs. Free WCAgs, PBS vs. IFA-WCAgs, Sham liposomes vs. Lip-WCAgs; ^●^ *p* < 0.05, Free WCAgs vs. IFA-WCAgs, ^●●^ *p* < 0.01, Free WCAgs vs. Lip-WCAgs. The values are shown as the means ± SD.

We assessed the prophylactic efficacy of vaccine formulations against *A. baumannii* infection in a mouse model. Immunization with Lip-WCAgs was highly effective and the immunized mice showed an 80% survival rate on day 30 post-*A. baumannii* infection (Figure 7A). Whereas, the mice in the IFA-WCAgs group had a 50% survival rate. All the mice immunized with Free WCAgs died within 30 days post-*A. baumannii* infection. The data showed that immunization with Lip-WCAgs and IFA-WCAgs significantly increased the survival rate as compared to the immunization with Free WCAgs (*p* = 0.0004 and *p* = 0.0006).

The severity of *A. baumannii* infection was assessed by analyzing the bacterial load in the lung tissues of the mice (Figure 7B). The mice in the PBS and sham liposomes injected groups had 566,152 ± 112,211 and 568,537 ± 72,881 CFUs/g of lung tissue, respectively (Figure 7B). Mice immunized with Lip-WCAgs had the lowest CFUs of 5464 ± 1193 per gram of lung tissue, which was significantly greater than the 237,126 ± 46,633 CFUs in the lung tissue of mice immunized with Free WCAgs (*p* < 0.01). On the other hand, the mice immunized with IFA-WCAgs had 51,521 ± 8066 CFUs, which was significantly lower compared to the 237,126 ± 46,633 CFUs in the lung tissues of the mice immunized with Free WCAgs (*p* < 0.05).

### 3.9. Lip-WCAgs-Immunized Mice Showed Lower Levels of Inflammation Markers

**Figure 8:**
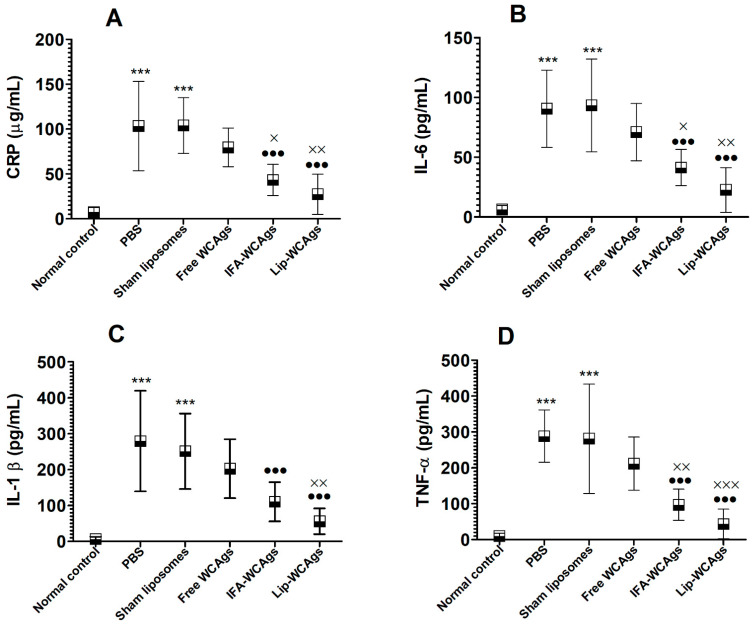
Lip-WCAgs-immunized mice showed the lowest level of CRP, IL-6, IL-1β and TNF-α in the systemic circulation. (**A**) CRP. *** (*p* < 0.001) normal control vs. PBS or Sham liposomes, ^●●●^ (*p* < 0.001) PBS vs. IFA-WCAgs or Lip-WCAgs, ^×^ (*p* < 0.05) Free WCAgs vs. IFA-WCAgs, ^××^ (*p* < 0.01) Free WCAgs vs. Lip-WCAgs. (**B**) IL-6. *** (*p* < 0.001) normal control vs. PBS or Sham liposomes, ^●●●^ (*p* < 0.001) PBS vs. IFA-WCAgs or Lip-WCAgs, ^×^ (*p* < 0.05) Free WCAgs vs. IFA-WCAgs, ^××^ (*p* < 0.01) Free WCAgs vs. Lip-WCAgs. (**C**) IL-1β. *** (*p* < 0.001) normal control vs. PBS or Sham liposomes, ^●●●^ (*p* < 0.001) PBS vs. IFA-WCAgs or Lip-WCAgs, ^××^ (*p* < 0.01) Free WCAgs vs. Lip-WCAgs. (**D**) TNF-α. *** (*p* < 0.001) normal control vs. PBS or Sham liposomes, ^●●●^ (*p* < 0.001) PBS vs. IFA-WCAgs or Lip-WCAgs, ^××^ (*p* < 0.05) Free WCAgs vs. IFA-WCAgs, ^×××^ (*p* < 0.01) Free WCAgs vs. Lip-WCAgs.

CRP is an important inflammatory marker in bacterial infectious diseases. The mean level of CRP was found to be 7.9 ± 1.5 µg/mL in the blood of normal mice was increased to 103 ± 20 µg/mL in PBS-injected *A. baumannii*-infected mice (Figure 8A) (*p* < 0.001). Mice immunized with IFA-WCAgs and Lip-WCAgs had significantly lower levels of CRP, 43 ± 7 and 27 ± 9 µg/mL, respectively (*p* < 0.001). Whereas, the mice immunized with Free WCAgs had 80 ± 8.6 µg/mL of CRP, which was significantly lower than the CRP level in IFA-WCAgs and Lip-WCAgs-immunized mice (*p* < 0.05 and *p* < 0.01, respectively). Moreover, IL-6, IL-1β, and TNF-α are other pro-inflammatory cytokines that are elevated in *A. baumannii*-infected mice (Figure 8B–D). The amount of IL-6 was found to be 6.2 ± 1.2 pg/mL in normal mice and was increased to 91 ± 13 pg/mL in PBS-injected *A. baumannii*-infected mice (Figure 8B) (*p* < 0.001). Immunization with Free WCAgs was not effective in alleviating the IL-6 level, which was found to be 71 ± 9.6 pg/mL in *A. baumannii*-infected mice. Interestingly, Lip-WCAgs-immunized mice had the lowest IL-6 level (23 ± 7.6 pg/mL) as compared to the IL-6 level in the blood of Free WCAgs-immunized mice (*p* < 0.001). Like IL-6, the level of IL-1β was highly raised in PBS-injected mice (Figure 8C). The amount of IL-1β was found to be 6.3 ± 2.5 pg/mL in the normal mice which was enhanced to 280 ± 56 pg/mL in PBS-injected mice (Figure 8C). Mice immunized with Lip-WCAgs had 56 ± 14 pg/mL of IL-1β which is significantly lower than the 280 ± 56 pg/mL of IL-1β level in PBS-injected or 203 ± 33 pg/mL in Free WCAgs-immunized mice (*p* < 0.01 and *p* < 0.001, respectively). TNF-α is an important pro-inflammatory cytokine that contributes to the pathogenesis of *A. baumannii*. TNF-α was found to be 281 ± 61 pg/mL in Sham-liposome immunized *A. baumannii*-infected mice as compared to the 10 ± 1.86 pg/mL normal value (Figure 8D) (*p* < 0.001). Interestingly, Lip-WCAgs-immunized mice had a TNF-α amount of 44 ± 9.6 pg/mL as compared to 97 ± 17 pg/mL in IFA-WCAgs-immunized, and 212 ± 30 pg/mL in Free WCAgs-immunized mice (Figure 8D).

## 4. Discussion

The outcome of the present work is very encouraging as the mice immunized with Lip-WCAgs exhibited superior antigen-specific immune responses and the greatest resistance against *A. baumannii* without any toxic manifestations. The antisera from Lip-WCAgs-immunized mice remarkably inhibited the formation of biofilm and showed greater opsonophagocytic killing activity against *A. baumannii*. In addition, Lip-WCAgs-immunized mice had alleviated levels of CRP, IL-6, IL-1β, and TNF-α.

In contemporary years, the phenomenon of multidrug resistance has been extensively observed in the clinical isolates of *A. baumannii* [4,5]. Ineffectiveness of the presently available antibiotics has resulted in a greater mortality rate of *A. baumannii* infected patients. Moreover, currently used antibiotics such as colistin and vancomycin have been shown to exert significant toxicity in the treated patients [28]. Combination therapy has been suggested to increase the efficacy of colistin against Carbapenem-resistant *Acinetobacter baumannii* (CRAB). Katip et al., reported that meropenem increased the activity of colistin against CRAB without altering the drug-induced nephrotoxicity [29]. Vaccination is becoming an important strategy to mitigate antimicrobial resistance and in the current scenario, the formulation of an effective vaccine is imperative in order to protect the individuals against drug-resistant *A. baumannii*. Until now, no vaccine against *A. baumannii* has been advanced to the clinical stage. Liposomes can deliver the encapsulated antigens to the endocytic and cytoplasmic compartments of the antigen-presenting cell (APCs) resulting in the elicitation of both the humoral and cell-mediated immune responses [30,31]. Recently, we reported that a Middle East Respiratory Syndrome coronavirus papain-like protease (MERS-CoV-PL pro) encapsulated in liposomes elicited greater immune responses in mice [23].

*A. baumannii* antigens, including inactivated whole cells, OmpA, biofilm-associated protein (Bap), Ata, outer membrane complexes (OMCs) and outer membrane vesicles (OMVs) have been identified as potent immunogenic molecules to develop vaccines [32,33,34,35,36,37,38,39]. Some of these vaccine formulations have shown weak immunogenicity and safety issues that hinder their applications in the clinical setting. In the present study, liposomes were used as vaccine carriers and immunoadjuvants for whole cell antigens of *A. baumannii*. The results showed that immunization with Lip-WCAgs induced remarkably greater activation of antigen-specific immune responses and effectively protected the mice against the systemic infection of *A. baumannii*. The safety of a vaccine is an important issue that should be addressed before bringing the vaccine for human use. Importantly, Lip-WCAgs did not induce any toxicity, as revealed by the parameters of AST, ALT, BUN, and LDH in immunized mice. Earlier studies showed that antibodies specific to outer membrane proteins of *A. baumannii* exhibited bactericidal and opsonizing activities [40]. Here, we showed that mice immunized with Lip-WCAgs had higher antigen-specific antibody levels as compared to the mice immunized with IFA-WCAgs or Free WCAgs. Interestingly, vaccination with Lip-WCAgs induced greater secretion of the IgG2a isotype as compared to the IgG1 isotype. IgG2a has been reported to exert superior neutralizing potential through the activation of effector immune responses [23]. Furthermore, the secretion of IgG2a can contribute to the proliferation of antigen-specific T cell responses [41]. This is also supported by the findings of the present study that showed the greater proliferation of lymphocytes from the mice immunized with Lip-WCAgs as compared to the lymphocytes from the mice immunized with IFA-WCAgs or Free WCAgs. IgG2a can shift the immune response in the favor of Th1 type which is also indicated by the presence of higher levels of Th1 cytokines in the mice immunized with Lip-WCAgs. Biofilm formation is an important virulence factor of *A. baumannii* that favors the survival of bacteria under unfavorable conditions and contributes to drug resistance. Biofilm formation depends on the outer membrane protein A (*omp*A) and pili, which contributes to the adherence of the biofilm to the surface. Importantly, the data of the current study demonstrated that antisera from the Lip-WCAgs-immunized mice substantially decreased the biofilm formation by *A. baumannii*. It suggests that antibodies have the ability to inhibit biofilm formation by *A. baumannii*. Earlier studies showed that polyclonal antibodies against fimbriae protein inhibited biofilm formation in about 80% of strains of *A. baumannii*, including the ATCC 19,606 used in the current study [42]. Moreover, a study by Erami et al., also documented the protective effects of anti-OMP antibodies against *A. baumannii*-induced sepsis in mice [43]. A study by Huang et al., showed that antibodies against anti-outer membrane vesicles (AbOMVs) can increase the activity of an antibiotic against drug-resistant *A. baumannii* [44]. Moreover, the results of the present study suggested that antisera from Lip-WCAgs-immunized mice showed the highest opsonophagocytic killing activity of macrophages against *A. baumannii*. It may be attributed to the presence of higher levels of IgG2a, which has superior neutralizing potential and may contribute to the clearance of the pathogen from the system. Some earlier studies have also reported that the administration of antigen-specific antibodies can increase the opsonophagocytic killing of *A. baumannii* which contributes to a higher survival time of the infected mice [36,45].

Here, the efficacy of various vaccine formulations was analyzed in the protection studies in a murine model against *A. baumannii*. Like its effect on immune response, immunization with Lip-WCAgs imparted the highest protection against *A. baumannii*. Mice immunized with Lip-WCAgs had an 80% survival rate on day 30 post-infection, whereas those immunized with IFA-WCAgs had a 50% survival rate. However, all the mice immunized with Free WCAgs died within day 30 post-infection. The bacterial load data in the lungs of the mice were found to be in agreement with the survival data as Lip-WCAgs-immunized mice had the lowest bacterial load in the lung tissues. The severity of *A. baumannii* infection was analyzed by measuring the levels of inflammation markers in the systemic circulation of mice immunized with Lip-WCAgs or IFA-WCAgs or Free WCAgs. We earlier showed that *A. baumannii*-infected mice had greater levels of CRP, IL-6, IL-1β, and TNF-α, which contribute to apoptosis in the lung tissues [29,46]. Interestingly, the mice immunized with Lip-WCAgs had the lowest levels of inflammatory markers in their systemic circulation as compared to the mice immunized with IFA-WCAgs or Free WCAgs. This may be ascribed to a highly reduced bacterial load and severity of the infection in the lung tissues of the mice immunized with Lip-WCAgs as compared to those immunized with Free WCAgs and IFA-WCAgs. A study by Huang et al., demonstrated that immunization with outer membrane vesicles (OMVs) of *A. baumannii* adjuvanted with alum significantly reduced the infiltration of inflammatory cells and the levels of inflammatory cytokines in bronchoalveolar lavage fluids [47]. However, they did not report the survival data of the infected mice. McConnell and Pachon explored the efficacy of the active and passive immunization with inactivated whole cell vaccine against *A. baumannii* infection-induced disseminated sepsis [33]. However, the use of inactivated whole cells may induce some toxicity due to the presence of lipopolysaccharides (LPS).

## 5. Conclusions

In summary, the findings of this study demonstrated that the immunization with liposome-encapsulated WCAgs of *A. baumannii* did not induce any toxicity, but it induced superior WCAgs-specific immune responses in mice. Moreover, the splenocytes from the mice immunized with Lip-WCAgs produced greater amounts of IFN-γ and IL-12, which can activate cell-mediated immune responses against pathogens. Notably, the mice immunized with Lip-WCAgs showed the highest resistance against the systemic infection of *A. baumannii* as shown by the higher survival rate and lower bacterial load. In addition, Lip-WCAgs-immunized mice had lower levels of inflammation markers, including CRP, IL-6, IL-1β, and TNF-α in the systemic circulation. Thus, Lip-WCAgs may serve as a prospective prophylactic vaccine formulation to protect against *A. baumannii* infection.

## Data Availability

All relevant data have been provided within the manuscript.
